# Profiling ambivalence in the context of nonsuicidal self‐injury

**DOI:** 10.1002/jclp.23494

**Published:** 2023-02-25

**Authors:** Nicole Gray, Hannah Uren, Ethan Pemberton, Mark Boyes

**Affiliations:** ^1^ School of Population Health Faculty of Health Sciences Curtin University Perth Western Australia; ^2^ School of Arts and Humanities Faculty of Psychology Edith Cowan University Perth Western Australia; ^3^ Curtin enAble Institute, Faculty of Health Sciences Curtin University Perth Western Australia

**Keywords:** ambivalence, behavior, desire, nonsuicidal self‐injury, profiles

## Abstract

**Background:**

We aimed to identify profiles of ambivalence among individuals with a history of non‐suicidal self‐injury (NSSI) and tested whether profiles differed across various theoretically informed constructs: NSSI‐related characteristics, cognitive (outcome expectancies, self‐efficacy to resist NSSI), emotional (psychological distress, difficulties in emotion regulation), personality, and incentives to engage/not engage in NSSI.

**Methods:**

Individuals with a lifetime history of NSSI (*n* = 224) reported the extent to which they wanted to and did not want to engage in NSSI and completed well‐validated measures of the constructs of interest.

**Results:**

Latent profile analysis indicated four ambivalence profiles (avoid: *n* = 39; moderately ambivalent: *n* = 85; highly ambivalent: *n* = 30; approach: *n* = 70). The profiles differed across a number of NSSI‐related characteristics, cognitive, emotional, and incentive‐related variables. Differences between the ambivalence profiles and the avoid/approach profiles varied across constructs. For example, the ambivalence and approach profiles were similar for NSSI‐related outcome expectancies, but the ambivalence and avoidance profiles were similar for self‐efficacy to resist NSSI.

**Conclusion:**

Findings highlight variation between the desire to engage or not engage in NSSI that are consistent with the notion of ambivalence. Understanding these differences may allow for a more person‐centered approach in treatment for NSSI.

## 1 INTRODUCTION

Non‐suicidal self‐injury (NSSI) is defined as the deliberate damage to one's own body tissue without suicidal intent (International Society for the Study of Self‐injury, 2018). Self‐injurious behaviors include cutting and burning the skin (Klonsky & Glenn, 2009). Clinical prevalence of the behavior is approximately 20% among adults, and 40%–80% among adolescents (Klonsky & Muehlenkamp, 2007). Community prevalence is approximately 13% among young adults aged 18–24, and 17% among adolescents aged 10–17. Among university samples, NSSI prevalence reaches approximately 20% (Swannell et al., 2014). Individuals report engaging in NSSI as a form of self‐punishment, antidissociation, and to regulate unwanted emotions (Taylor et al., 2018). Although not suicidal in nature, NSSI may be associated with future suicidal thoughts and behaviors and other psychopathology, potentially worsening over time (Kiekens et al., 2018). A highly stigmatized behavior, self‐injury may lead to fear of judgment, and ongoing shame when one discloses their behavior, possibly perpetuating further self‐injury (Staniland et al., 2020). While engagement in NSSI may lead to negative outcomes and distress, many individuals who engage in the behavior also report benefits, including emotional relief, the ability to communicate distress, and expressions of strength. As such, there may be continued engagement in NSSI despite the associated negative outcomes. Recent findings suggest that approximately 20% of individuals with a history of self‐injury do not want to stop the behavior (Gray et al., 2022).

Current theories of self‐injury focus on the behavior itself; typically attempting to explain the likelihood of engaging in NSSI or not (see Hasking et al., 2017 for a review). However, it may be beneficial to consider the step before engagement in self‐injury—the desire to self‐injure (Gray et al., 2022). Desire as a preceding factor to behavior has long been acknowledged in the substance use literature (Breiner et al., 1999). The substance use literature recognizes that individuals may consciously desire one thing, while simultaneously holding a competing desire; referred to as ambivalence in craving (Breiner et al., 1999; Schlauch et al., 2015). The Ambivalence Model (Breiner et al., 1999) proposes that the desire to engage in a behavior (approach) or avoid engaging in the behavior (avoid) may both exist on a continuum. The model includes historical factors (e.g., reactivity, personality, and past reinforcement); immediate factors (incentives); and outcome expectancies. The interaction of these factors may generate an in‐the‐moment level of desire to, and not to engage in substance use (Breiner et al., 1999). According to the model, these preceding factors will lead to varying inclinations for craving, conceptualized into four quadrants—avoid, moderately ambivalent, highly ambivalent, and indifferent (Breiner et al., 1999). Schlauch and colleagues (2015) validated the concept of ambivalence among individuals who engage in substance use, profiling their sample into five groups; indifferent, approach, avoid, moderately ambivalent, and highly ambivalent. Additionally, approach and highly ambivalent profiles engaged in more drinking, and more negative outcomes in comparison to other profiles. In contrast, participants with avoidance and ambivalent inclinations were more likely than other profiles to have admitted themselves into a substance use treatment program (Schlauch et al., 2015). While the concept of ambivalence is well‐studied in the area of substance use, its potential for aiding the understanding NSSI has only recently been proposed (Gray et al., 2021).

The Ambivalence Model postulates that difficulty with emotion, reasons to engage/not engage, psychological well‐being, self‐confidence, expectations of both desired and undesired outcomes, previous reinforcement, and personality traits could be associated with varying levels of ambivalence (Breiner et al., 1999). These factors are all also associated with NSSI (Hasking et al., 2017), although these studies examined associations with self‐injurious behaviors rather than desire.

Experiences consistent with ambivalence have been reported across a range of NSSI literature (Gray et al., 2021). An individual may experience ambivalence during the engagement of self‐injury, whereby they want to engage in the behavior to relieve pain or negative affect, yet do not want to engage in the behavior out of fear of attracting unwanted attention (Gray et al., 2021). In previous work comparing individuals who have, and have not stopped self‐injury, there were many differences across a range of cognitive and emotional variables (NSSI functions, psychological distress, difficulties with emotion regulation, outcome expectancies, and self‐efficacy). Individuals who had stopped engaging in self‐injury used the behavior less for intrapersonal reasons compared to individuals who had not stopped. Additionally, individuals who had stopped showed less psychological distress, less difficulties with emotion regulation, less pain expectancies through NSSI, and greater self‐efficacy to resist the behavior, compared to individuals who had not stopped (Gray et al., 2022). However, this same study found very few differences in the same variables when comparing individuals who wanted to and did not want to stop self‐injuring. This may be because participants were given a binary no/yes response option when asked if they wanted to stop self‐injury, potentially not capturing the concept of ambivalence.

**Figure 1:**
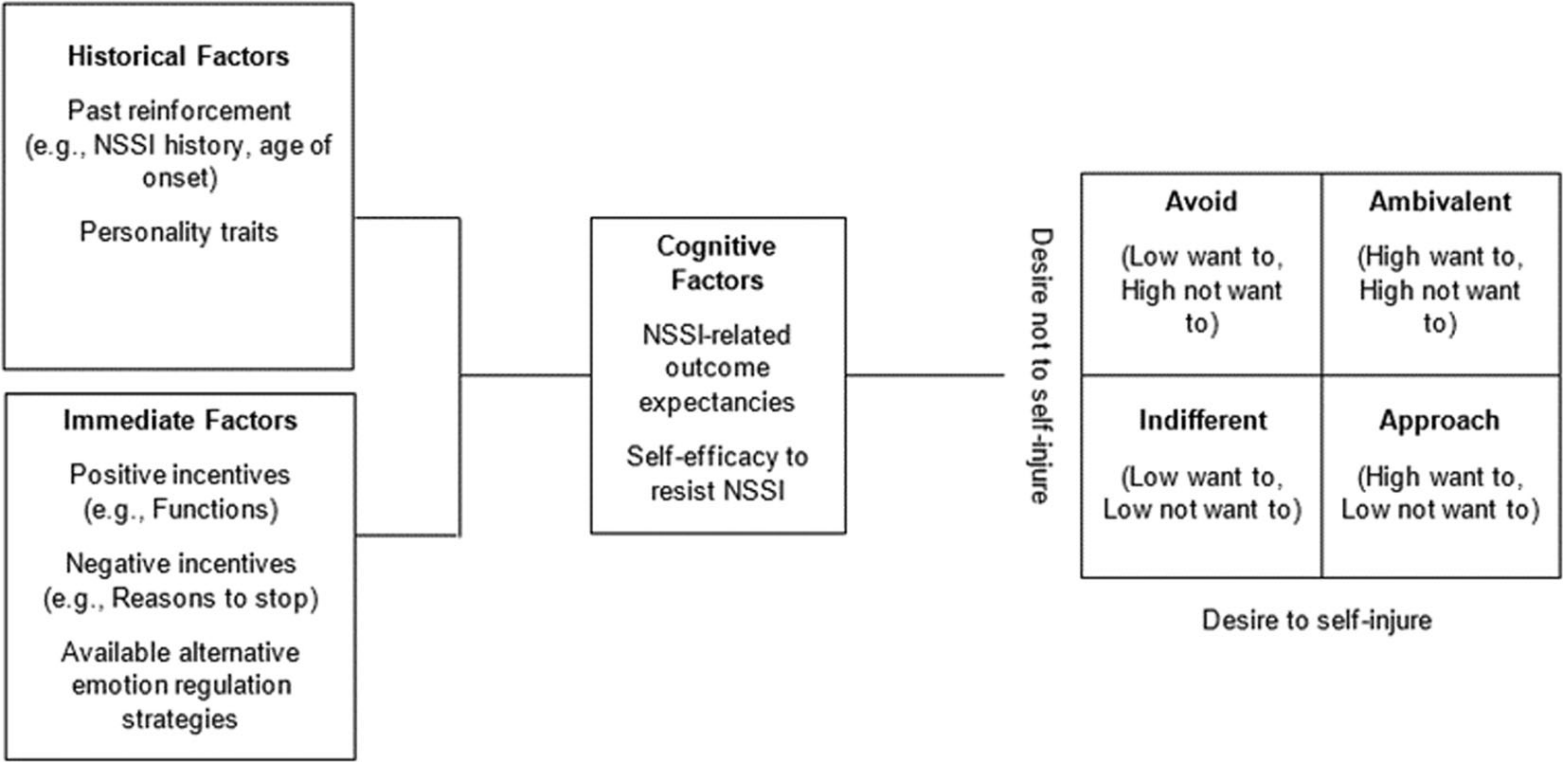
An application of the ambivalence model to self‐injury.

The current study sought to build on this previous finding to (i) determine whether profiles of ambivalence in the desire to self‐injure can be identified and (ii) whether these potential profiles differ on a range of theoretically informed constructs. Using the Ambivalence Model (Breiner et al., 1999) as a framework, we examined ambivalence among individuals with a history of NSSI. Specifically, we examined the extent to which individuals with a history of NSSI hold competing desires to both want to, and not want to, self‐injury, and tested whether differences in these desires to self‐injure and not self‐injure could be used to generate profiles in accordance with the Ambivalence Model. We also explored whether the potential profiles differed across demographics, as well as the constructs included in the Ambivalence Model. We explored Historical factors: past re‐enforcement (NSSI, e.g., age of onset, history of engagement in NSSI, history of desire to engage in NSSI), and personality; Immediate factors: positive incentives (functions of self‐injury), negative incentives (reasons to stop self‐injury), available alternatives (difficulties with emotion regulation, psychological distress); and cognitive factors: outcome expectancies, self‐efficacy to resist NSSI. The variables we sought to examine were predicted to underpin the ambivalence experienced by individuals in our predicted profiles (Figure 1).

## 2 METHOD

### 2.1 Participants

Responses to an online survey were collected from a combined community and university student sample. A range of recruitment methods was used including snowball sampling through the general community, and the Curtin University undergraduate participation pool. Of the 224 participants, 35 (15.6%) identified as male, 167 (74.6%) identified as female, and 22 (9.8%) identified as another gender (transgender, nonbinary, or another unspecified gender). Participants were between 17 and 39 (*M* = 21; SD = 4.43). One hundred and forty‐six (65.2%) were university students. One hundred and three participants (46%) were born in Australia. Approximately 59% were living in Australia, 31% were living in other countries, and 10% of participants did not specify their location. All participants had engaged in NSSI at some point in their lives. The mean age for initial engagement in NSSI was 14 years (SD = 3.05). Cutting was the most common method of NSSI (*n* = 146; 66.1%), followed by self‐battery (*n* = 17; 7.6%), biting (*n* = 10; 4.5%), and pinching (*n* = 10; 4.5%). Most participants reported feeling pain when self‐injuring (*n* = 218; 97%).

### 2.2 Measures

#### 2.2.1 Desire to self‐injure and not self‐injure

The extent to which participants wanted to self‐injure and not self‐injure was assessed with two items: “To what extent have you wanted to self‐injure over your lifetime?”; “To what extent have you not wanted to self‐injure over your lifetime?.” Responses were made on a 10‐point scale (1: *only slightly*; 11: *very much*).

#### 2.2.2 Tendency to approach/avoid NSSI

Tendencies to approach or avoid NSSI were assessed using an adapted version of the Brief Approach Avoid Alcohol Questionnaire ([BAAAQ]; Levine et al., 2019). Participants were asked the extent to which they would have “liked to engage in NSSI” in the past year. The original version has demonstrated sound internal consistency in the alcohol‐use literature (Levine et al., 2019) and reliability was good in our sample (approach: *α* = 0.92; avoid: *α* = 0.86).

#### 2.2.3 NSSI characteristics and functions

Self‐injury history, characteristics, and functions were assessed using the Inventory of Statements about Self‐injury (ISAS; Klonsky & Glenn, 2009). The ISAS consists of two sections; Section I provides a definition of NSSI and asks participants to respond to items about their history with self‐injury including frequency, recency, age of onset, and methods. Section II assesses 13 functions of self‐injury. These functions are divided into two higher‐order subscales: interpersonal and intrapersonal functions. Internal consistency in the current sample was acceptable across higher‐order subscales (intrapersonal: *α* = 0.85; interpersonal: *α* = 0.89) and individual subscales (interpersonal influence: *α* = 0.66; antisuicide: *α* = 0.89).

#### 2.2.4 Personality

Personality was assessed using the Mini Interpersonal Personality Item Pool ([IPIP]; Donnellan et al., 2006), which assess five personality traits; extraversion, agreeableness, conscientiousness, neuroticism, and intellect/imagination. Reliability ranged between *α* = 0.65 (neuroticism) and *α* = 0.84 (extraversion) in our sample.

#### 2.2.5 Reasons to stop self‐injury

Reasons to stop self‐injury were measured by the Reasons to Stop Self‐injury Questionnaire (Turner et al., 2014), which comprises nine subscales: Desire for Change/Resolution of Distress; Situational and Environmental Deterrents; Negative Emotional Consequences; Fear of Discovery and Stigma; Negative Impact on Relationships; Addiction to NSSI; Others’ Expectations; Negative Physical Consequences; Body Concerns. Reliability ranged between *α* = 0.75 (Situational and Environmental Deterrents) and *α* = 0.87 (Desire for Change/Resolution of Distress) in our sample.

#### 2.2.6 Difficulties in emotion regulation

Difficulties in emotion regulation were assessed using the 18‐item Difficulties in Emotion Regulation Scale (Victor & Klonsky, 2016). Difficulties in emotion regulation may be evaluated as an overall construct or six individual subscales: limited awareness of emotion; difficulties clarifying emotional experiences; difficulties with goal‐oriented behaviors; difficulties managing impulsive behaviors; nonacceptance of emotions; difficulties accessing regulation strategies. Reliability in the current sample was excellent for the overall measure (*α* = 0.89), and good‐excellent for each of the subscales (strategies: *α* = 0.83; goals: *α* = 0.93).

#### 2.2.7 Psychological distress

Psychological distress were assessed using the 10‐item version of the Kessler Psychological Distress Scale (Kessler et al., 2002), which assesses the frequency of psychological distress symptomology over the previous 4 weeks. Internal consistency was excellent in our sample (*α* = 0.89).

#### 2.2.8 NSSI‐related outcome expectancies

Self‐injury‐related outcome expectancies were assessed using the Nonsuicidal Self‐Injury Expectancies Questionnaire (Hasking & Boyes, 2018). The measure comprises five subscales (affect regulation, negative social experiences, communication, pain, and negative self‐beliefs) assessing participants’ perceived likelihood of a given outcome when engaging in self‐injury. In the current sample, internal consistencies were adequate (pain: *α* = 0.72; negative social outcomes: *α* = 0.82).

#### 2.2.9 Self‐Efficacy to Resist NSSI

Assessment of participants’ perceived ability to resist engaging in self‐injury was completed using the Self‐Efficacy to Resist Nonsuicidal Self‐Injury scale (SERN; Dawkins et al., 2022). The SERN comprises three subscales assessing self‐efficacy to resist NSSI across different contexts; where there is a greater risk of engaging in the behavior, due to difficult internal states (risk contexts), where there are protective factors possibly making it easier to resist the behavior (protective contexts), and contexts where there are reminders of self‐injury (reminder contexts; Dawkins et al., 2022). Internal consistency for all subscales was excellent in our sample (Risk: *α* = 0.93; Protect: *α* = 0.90; Reminders: *α* = 0.95).

### 2.3 Procedure

After gaining ethical approval from Curtin University HREC the study was advertised on the university online participant pool, and on social media platforms. Participants were directed to a survey hosted by Qualtrics. The questionnaire took approximately 45–60 min to complete. Participants from the Curtin University participant pool university participant pool received course credit for participation, while participants from outside the university were placed into a draw to receive 1 of 20 $50 e‐gift cards. Participants received information and contact information for NSSI support services on completion of the survey.

### 2.4 Data analysis strategy

Analyses were conducted in five stages. First, a missing values analysis was conducted. Participants who did not answer the questions “To what extent have you wanted to self‐injure in your lifetime” and “To what extent have you not wanted to self‐injure in your lifetime” were excluded from the analysis. Missing values analysis revealed that data were missing completely at random for the K10 (*χ* ^2^(27) = 26.07 *p* = 0.514), the Mini IPIP (*χ* ^2^(179) = 198.15, *p* = 0.16), and the Reasons to Stop Self‐injury Questionnaire (*χ* ^2^(813) = 832.87, *p* = 0.31). Although the data across other measures were not missing completely at random, there was minimal missing data (<2%), therefore, expectation maximization was used to impute all remaining missing data. Second, individual differences in the desire to stop and not stop self‐injuring were explored. Third, a latent profile analysis (LPA) was conducted. LPA is a person‐centered modeling technique designed to identify groups (i.e., profiles) of people that share a similar pattern of responses across a set of variables. The variables of interest included the extent to which one has wanted to and not wanted to self‐injure over their lifetime. Finally, to further validate the profiles, profile‐related differences on the approach and avoidance subscales of the adapted BAAAQ, as well as variables in the Ambivalence Model (past re‐enforcement, personality, positive incentives, negative incentives, available alternatives, outcome expectancies, self‐efficacy to resist NSSI) were analyzed. *χ* ^2^ analyses were conducted for categorical variables and multivariate analysis of variances with appropriate univariate follow‐up tests were conducted on conceptually grouped scale variables. Due to the exploratory nature of the study, no covariates were included in the analyses. Statistical significance was set at *α* ≤ 0.05.

## 3 RESULTS

All participants in the sample reported a lifetime history of engagement in NSSI. Except for one participant, all participants reported that they had felt the desire to self‐injure in their lives; 83% of participants reported wanting to engage in NSSI the last year. Seventy‐seven percent of participants had engaged in NSSI in the last year. Of those, 51% had done so on more than 5 days in the last year. Seventy‐five percent of participants reported wanting to engage in NSSI in the last month. Forty‐six percent of participants had engaged in NSSI in the last month. Of those, 46% had done so on more than 5 days in the last year. Eighty‐four percent reported wanting to stop self‐injury at some point in their lives, and 16% of individuals reported no desire to stop self‐injuring.

### 3.1 Profiling ambivalence among individuals with a history of self‐injury

An LPA was conducted using the TidyLPA package with *R* Studio software (Rosenberg et al., 2019). The chosen model had equal variances across profiles, and covariances fixed to zero. Solutions for 1–12 profiles were tested. The optimal profile solution was evaluated against a set of statistical criteria. This included five common fit indices: the Akaike Information Criterion (AIC), Bayesian Information Criterion (BIC), Classification Likelihood Criterion (CLC), Kullback Information Criterion (KIC), and Appropriate Weight of Evidence Criterion (AWE), and the Bootstrap Likelihood Ratio Test (BLRT). For each of these fit indexes, values closer to 0 indicate a better‐fitting model. An Entropy index (i.e., the estimation of classification accuracy) was also calculated for each model. Values (ranging 0–1) closer to 1 indicate higher levels of statistical certainty pertaining to the extracted profiles, whereby values of 0.80 or more are considered acceptable (Tein et al., 2013). Profile size was also considered when determining the optimal profile solution; profiles containing less than 5% of the total sample are typically considered insignificant (Nasserinejad et al., 2017) and excluded when considering the optimal solution. Finally, the models were evaluated with reference to how theoretically relevant and distinct the extracted profiles were (Foti et al., 2012).

Models with more than seven profiles extracted at least one profile containing less than 5% of the total sample and were therefore excluded (Nasserinejad et al., 2017). While most of the fit indices demonstrated the lowest values for a six‐profile solution, the BIC value was lowest for a four‐profile solution The BIC is the most utilized indicator of a suitable profile. As such, this index was used as our focus for fit (Spurk et al., 2020). Additionally, the four‐profile solution contained close to twice the sample size in the smallest profile compared to a six‐profile solution. Entropy was within the acceptable range (Supporting Information: Table S1) for a six‐profile solution; however, a four‐profile solution was approaching acceptable parameters (0.78; Tein et al., 2013). The four‐profile solution was theoretically more meaningful and parsimonious, which was evaluated when distinguishing appropriate profiles (Foti et al., 2012). A four‐profile solution also corresponds to the already existing literature on ambivalence (Breiner et al., 1999) and was deemed the most appropriate.

**Figure 2:**
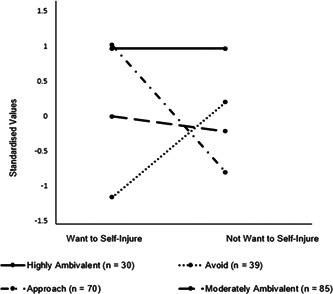
Ambivalence profiles.

The four‐profile extraction demonstrated distinct profiles differing on the level of desire to avoid and approach self‐injury (see Figure 2). Profile 1 (highly ambivalent; *n* = 30; 13.4%) reported high levels of wanting to self‐injure, and high levels of not wanting to self‐injure throughout their lifetime. Profile 2 (avoid; *n* = 39; 17.4%) reported low levels of wanting to self‐injure, and high levels of not wanting to self‐injure throughout their lifetime. Profile 3 (approach; *n* = 70; 31.3%) reported high levels of wanting to self‐injure, and low levels of not wanting to self‐injure throughout their lifetime. Profile 4 (moderately ambivalent; *n* = 85; 37.9%) reported midway levels of wanting to self‐injure, and midway levels of not wanting to self‐injure throughout their lifetime.

### 3.2 Validation of profiles

Individuals in the approach profile scored higher on thoughts and desire toward NSSI than individuals who experienced any ambivalence (high and moderate ambivalence profiles), and higher than individuals in the avoid profile (Supporting Information: Table S2). No differences were found between higher and moderate ambivalence profiles. Individuals who reported a stronger desire to not engage in NSSI (avoid profile) scored lowest on thoughts and desire toward NSSI compared to all other profiles. Group differences were also found in levels of thoughts and desire away from NSSI; individuals who experienced less desire to engage in NSSI, and a stronger desire to not engage in NSSI (avoid profile) scored lower on thoughts and desire away from NSSI than the other profiles (approach, high ambivalence, moderate ambivalence; Supporting Information: Table S2).

### 3.3 Profile‐related differences on historical factors

Exploring profile‐related differences in NSSI characteristics allowed further illustration of the utility of the ambivalence profiles. We compared more recent (past month) desire to self‐injure across the four profiles. Those who did not want to self‐injure in the last month had the highest proportion within the avoid profile (*n* = 56.5%), followed by the moderately ambivalent profile, followed by the highly ambivalent profile, with the lowest proportion in the approach profile (*n* = 3%; Supporting Information: Table S3). Additionally, the profiles were validated using a measure that asks for thoughts and desires around NSSI in the year prior. Profiles extracted on lifetime ambivalence correspond meaningfully with responses on past year ambivalence and reported desire over the month prior.

We also tested differences in NSSI characteristics across profiles. The mean age of the individuals in the avoid profile were significantly older than the individuals in the approach profile. The age of onset of NSSI was significantly younger in the approach profile, compared to the moderately ambivalent, and avoid profiles (Supporting Information: Table S2). Some differences were found in personality traits between profiles. The avoid profile showed significantly higher levels of extraversion than the other three profiles. The approach profile reported higher levels of neuroticism compared to the avoid and moderately ambivalent profiles, though not significantly different to the highly ambivalent profile.

### 3.4 Profile‐related differences on immediate factors

Several profile‐related differences in NSSI function were identified (Supporting Information: Table S2). Individuals in the approach and ambivalent profiles reported more intrapersonal reasons (e.g., affect regulation) than individuals from the avoid profile. No differences were found in interpersonal functions, except engaging in NSSI for self‐care (Supporting Information: Table S2).

The approach profile reported less reasons to stop engaging in NSSI than they avoid, and moderately ambivalent profiles. Specifically, they reported higher perceived addictive qualities of NSSI, less desire for change/resolution of distress, less perceived negative emotional consequences, and less body concerns than the avoid and moderately ambivalent profile. No significant differences were found between the approach and the highly ambivalent profile, except for their desire for change; compared to other profiles, individuals from the approach profile reported less desire for change.

Individuals in the approach profile reported more difficulties with emotion regulation than all other profiles. In comparison to the other three profiles, the approach profile reported more difficulty with awareness and clarity of their emotions, more difficulty with impulse control, more difficulty accepting their emotions, and more difficulty implementing strategies to regulate emotion. Individuals in the avoid profile and ambivalent profile responded similarly across all areas of emotion regulation, except for difficulties with emotion regulation strategies; here the ambivalent profiles reported similar difficulties with emotion regulation strategies, more difficulties than the avoid profile, and less difficulties than the approach profile. Additionally, individuals who tend to approach NSSI reported higher levels of psychological distress than those who tend to avoid the behavior, or experience ambivalence (Supporting Information: Table S2).

### 3.5 Profile‐related differences on cognitive factors

Profiles differed on certain outcome expectancies related to engaging in NSSI (Supporting Information: Table S2). Specifically, the approach and ambivalence profiles in comparison to the avoid profile reported expecting more affect regulation to occur through NSSI. Individuals in the approach profile reported expecting more affect regulation than individuals who were moderately ambivalent, though showed similar expectancies as the highly ambivalent profile. Individuals in the avoid, moderate, and highly ambivalent profiles reported significantly higher self‐efficacy to resist NSSI compared to individuals in the approach profile.

## 4 DISCUSSION

The purpose of this study was to examine if individuals with a history of NSSI differ in the level to which they both want to and do not want to self‐injure, and whether these differences in desire could be used to generate profiles consistent with the Ambivalence Model. Additionally, our research explored whether these profiles differed across several theoretically informed constructs, including cognition, emotion, personality, and incentives to engage (and not engage) in NSSI.

Understanding ambivalence in the context of NSSI may be important to recovery (Gray et al., 2021; Kelada et al., 2017). Previous studies identified that individuals who had stopped self‐injuring used self‐injury less for intrapersonal functions and had less psychological distress, less difficulties with emotion regulation, less pain expectancies, and greater self‐efficacy to resist NSSI compared to individuals who had not stopped (Gray et al., 2022). However, these factors did not differentiate individuals who wanted/did not want to stop self‐injuring. We postulated that this may be because desire to engage in, or stop, a behavior is more complex than a dichotomous outcome of no or yes. Our current findings suggest that differences do exist when simultaneously considering competing desires.

Consistent with the substance use literature (Schlauch et al., 2015), the four‐profile solution found in the profile analysis comprised of avoid, moderately ambivalent, highly ambivalent, and approach profiles. Profile‐related differences on NSSI characteristics and the amended approach/avoidance questionnaire provided further support for these profiles. Members of the approach profile responded with the highest levels of approach tendencies, the ambivalent profiles responded with moderate levels, and the avoid profile responded with the lowest levels. One exception of our expectations was that the avoid profile scored lowest on the avoid subscale of the adapted BAAAQ (Levine et al., 2019) This could reflect the nature of some items. The avoidance subscale measures behavioral avoidance. For example, the item “I deliberately occupied myself so I would not self‐injure” would apply to those who want to self‐injure to some degree (i.e., ambivalent or approach profiles). As such, we might expect to see those with no desire to self‐injure reporting less tendency to avoid the NSSI.

The model underpinning this research (Breiner et al., 1999) describes an “indifferent” group, who have little to no desire to engage, and little to no desire not to engage in substance use. Our analyses did not indicate an indifferent group within our sample. One of the components of the Ambivalence Model is past reinforcement. Breiner and colleagues (1999) posit that desire to engage in behavior can come from biochemical reinforcement (i.e., a pleasurable feeling, or relief of an unwanted feeling), and from learning processes, where repetition leads to habitual reinforcement. The four profiles extracted in our study (avoid, moderately ambivalent, highly ambivalent, and approach) were appropriate for the sample, who all had a history of NSSI. More than half of the sample in each group had self‐injured in the previous year, and the group with the lowest number of individuals to have self‐injured in the last month were the avoid group. Consistent with research (Taylor et al., 2018) and the Ambivalence Model (Breiner et al., 1999), reinforcement was likely to be a factor surrounding the desire to self‐injure. In hindsight, given the nature of the sample, we believe this could account for the lack of a distinct group who felt indifferent toward the behavior.

There were differences between the avoid group and the approach group across several of the explored variables. This was most common among the cognitive‐emotional variables, such that individuals who approached NSSI had greater difficulties with emotion regulation, less self‐efficacy to resist NSSI, greater psychological distress, and greater affect regulation expectancies than the group who avoided NSSI. Additionally, there were differences between the avoid and approach profiles in affect regulatory incentives to approach (intrapersonal functions) such that the approach group reported engaging in NSSI to regulate affect, punish themselves, reduce dissociation, and avoid suicide to a greater extent than the avoid group. The differences found in this study are consistent with the literature, and cognitive‐emotional theories of NSSI, affirming that the behavior is most often utilized as an affect regulation strategy (Taylor et al., 2018).

Differences between avoid and approach were found among reasons to stop NSSI. However, this was only on items pertaining to personal feelings around their own self‐injurious behaviors (e.g., negative emotional consequences, loss of control) and not external factors (e.g., situational and environmental deterrents, others’ expectations). It is worth noting the variability between the profiles on the desire for change/resolution of distress as a reason to stop. The approach profile was significantly lower in their desire for change than the other three profiles. Our results are consistent with the literature in that wanting change/wanting to resolve distress is related to wanting to avoid/cease NSSI (Buser et al., 2014). In line with theories of behavior change, it is possible that highly ambivalent individuals are on the verge of change; with similar levels of desire toward NSSI, yet significantly different levels of desire for change compared to the approach group change (Grunberg & Lewis, 2015).

Theoretically, behavior change is underpinned by a desire to change (Grunberg & Lewis, 2015). This is where motivational interviewing techniques are considered beneficial, as the client explores the costs and benefits of a given behavior (Grunberg & Lewis, 2015). Given the clear profiles of ambivalence, and profile‐related differences in intrapersonal functions, with minimal differences in interpersonal functions, treatment targets that focus on the internal, emotional benefits of their behavioral desire may be beneficial in terms of resolving ambivalent states. This may include shame reduction, self‐efficacy, and self‐compassion if their desired behavior is to continue self‐injuring.

Additionally, numerous studies have assessed the reasons for self‐injury alongside the barriers to cessation (Buser et al., 2014; Kruzan & Whitlock, 2019). In these studies, interpersonal relationships are both a reason to stop (e.g., letting others down; Kruzan & Whitlock, 2019) and a mechanism for change (leaving unhealthy relationships/environments; Buser et al., 2014). While we did not identify profile‐related differences in interpersonal functions (reasons to self‐injure), it may be beneficial for future research to examine potential differences in interpersonal relationships (e.g., adverse family functioning, peer conflict, romantic relationship issues).

The moderately ambivalent group tended to report consistently middle‐range scores across variables. For most of the variables measured, very few differences were found between the moderate and highly ambivalent profiles; both ambivalence profiles tended to be similar across variables, with significant differences alternating between the avoid group and the approach group. In some cases, ambivalence appeared similar to the approach group (e.g., NSSI expectancies), other times ambivalence appeared similar to the avoid group (e.g., self‐efficacy to resist NSSI, difficulties with emotion regulation, psychological distress). Individuals experiencing ambivalence appear to fluctuate in their responses across cognitive emotional variables. Individuals who have high levels of wanting to engage in NSSI may state that they have no desire to self‐injure (highly ambivalent), while internal desires collide with conscious wishes to not self‐injure (Kelada et al., 2017). It is important for clinicians and other health professionals to acknowledge this while conducting emotional well‐being measures. Linking to our findings and using the widely used K‐10 scale (Kessler et al., 2002) as an example regarding psychological distress, individuals who report having high desire to avoid self‐injury may score similarly to individuals who strongly want to avoid *and* approach self‐injury. Without understanding and acknowledging ambivalence in treatment, client characteristics may be misread, hindering treatment. Yes or no framed questions to whether a client wants to self‐injure may only be capturing a small component of the more complex response. Acknowledging experiences of ambivalence in treatment may identify treatment targets for the clinician and a more person‐centered approach to therapeutic practices.

### 4.1 Theoretical and clinical implications

Our findings are consistent with current theories of emotion regulation, and its role in NSSI (Hasking et al., 2017). Our latent profile analyses show empirical evidence supporting the Ambivalence Model. NSSI models could benefit by considering that individuals may have conflicting desires toward their self‐injury and that these desires are likely important in understanding the behavior. Ambivalence not only impacts individuals who self‐injure but may cause frustration for health professionals who see recurring self‐injury during an extended recovery process (Saunders et al., 2012). Additionally, families may experience distress, anger, feelings of failure, confusion, and fear that their loved ones may re‐engage in the behavior (Kelada et al., 2017). For those desiring to cease NSSI, the acknowledgment that re‐engaging is not a failure, but rather a part of the broader, nonlinear recovery process has been identified as a valuable therapeutic approach (Gray et al., 2021). Levels of desire to engage/not to engage are likely to fluctuate when there are perceived advantages and disadvantages of any given behavior (Grunberg & Lewis, 2015). Health professionals would benefit from considering desire to change as a multidimensional construct. An individual can want to, and not want to self‐injure at the same time, and they may not be consciously aware of this conflict. Our findings also suggest that assessment of emotion, cognition, motivations, level of risk, and desire to change are not sufficiently capturing the individuals experience when accounting only for unidimensional responses. Compared to individuals who want to avoid self‐injury, psychological distress appears higher in those who want to approach the behavior. Additionally, psychological distress was higher in those who were ambivalent about self‐injury, compared to those who wished to avoid the behavior. Given this, if an individual state that they do not want to self‐injure anymore, it is not necessarily an indicator of improved psychological well‐being, particularly if they also hold a desire to continue the behavior.

### 4.2 Limitations and future research directions

The participants in our study were a community sample, largely university students. It may be of benefit to examine the nature of ambivalence in a clinical sample where rates of self‐injury are higher, and/or more recent. Due to a limited number of participants having had engaged in NSSI in the previous year, and previous month, our study was assessing ambivalence toward NSSI with a cross‐sectional data set using lifetime history of desire, while the measure that was used to validate the profiles asked for levels of avoid/approach over the past year. The disparity between lifetime and past year approach/avoid may have been problematic. Lifetime desire could capture fluctuations over time rather than simultaneous, competing desires. However, our profiles were further validated through measuring NSSI engagement in the past year and month. The highest within‐group percentage of NSSI engagement for the past year and month were approach, moderately ambivalent, highly ambivalent, and avoid, respectively, matching the levels of desire in our profiles. While this strengthens the validity of our profiles, it does not address experiencing competing desires simultaneously. Ecological momentary assessment studies could capture these competing desires in the moment and would provide a more rigorous assessment of ambivalence, as well as fluctuations in ambivalence and how these relate to self‐injurious behavior.

### 4.3 Conclusion

We established that ambivalence profiles can be identified in the context of NSSI, and that these profiles appear to differ meaningfully on a range of NSSI‐related, cognitive, emotional, and personality variables. In particular, individuals with approach or ambivalence tendencies report more psychological distress. Importantly, individuals who self‐injure may hold competing and seemingly contradictory desires (Kelada et al., 2017). Understanding this could have important theoretical and clinical implications. Acknowledging the possibility of ambivalence may reduce barriers to help‐seeking, as well as potentially improve clinician–client rapport, identify treatment targets, reduce confusion and/or shame, educate loved ones who may not understand the behavior, and increase client well‐being.

## AUTHOR CONTRIBUTIONS

Nicole Gray conceived and wrote the paper. Mark Boyes, Hannah Uren, and Ethan Pemberton reviewed and contributed to the writing of the paper.

## CONFLICT OF INTEREST STATEMENT

The authors declare no conflict of interest.

### PEER REVIEW

The peer review history for this article is available at https://www.webofscience.com/api/gateway/wos/peer-review/10.1002/jclp.23494.

## ETHICS STATEMENT

This study was approved by the Curtin University Human Research Ethics Committee. The research was conducted in accordance with the National Statement on the Conduct of Human Research and the Declaration of Helsinki. All participants provided consent to participate.

## Supporting information

Supporting information.

Supporting information.

Supporting information.

## Data Availability

The data that support the findings of this study are available on request from the corresponding author upon reasonable request. The data are not publicly available due to privacy or ethical restrictions.
