# Supplementary material for: Profiling ambivalence in the context of nonsuicidal self‐injury
Source: J Clin Psychol. 2023 Feb 25;79(8):1699–712. doi: 10.1002/jclp.23494 (PMC10952785; doi:10.1002/jclp.23494)
Supplement: Supplementary file 1 — Supporting information. [file JCLP-79-1699-s002.docx]

*Table S1. Fit and Entropy Index Values for One to Seven Profile Solutions*

| **Indices** | | | | | | | |
| --- | --- | --- | --- | --- | --- | --- | --- |
| **Number of profiles** | **AIC** | **BIC** | **CLC** | **KIC** | **AWE** | **Entropy** | ***N* min** |
| **One** | 1155 | 1169 | 1149 | 1162 | 1200 | 1 | 1.00 |
| **Two** | 1117 | 1141 | 1105 | 1127 | 1199 | .75 | .26 |
| **Three** | 1120 | 1154 | 1101 | 1133 | 1237 | .56 | .22 |
| **Four** | 1081 | 1125 | 1056 | 1097 | 1233 | .78 | .13 |
| **Five** | 1086 | 1140 | 1056 | 1105 | 1273 | .83 | .04 |
| **Six** | 1062 | 1127 | 1026 | 1084 | 1285 | .84 | .07 |
| **Seven** | 978 | 1053 | 936 | 1003 | 1237 | .83 | .05 |

*Note. AIC – Akaike Information Criterion; BIC – Bayesian Information Criterion; CLC – Classification Likelihood Criterion; KIC – Kullback Information Criterion; AWE – Appropriate Weight of Evidence Criterion; N min – Percentage in smallest group*
