# Supplementary material for: Profiling ambivalence in the context of nonsuicidal self‐injury
Source: J Clin Psychol. 2023 Feb 25;79(8):1699–712. doi: 10.1002/jclp.23494 (PMC10952785; doi:10.1002/jclp.23494)
Supplement: Supplementary file 2 — Supporting information. [file JCLP-79-1699-s003.docx]

| *Table S2. Descriptive statistics and group differences across each profile* | | | | | | | | | | |
| --- | --- | --- | --- | --- | --- | --- | --- | --- | --- | --- |
|  | **Avoid** ^a^ | **Mod Amb** ^b^ | **High Amb** ^c^ | **Approach** ^d^ | **Group Differences** | | | | |  |
|  | ***M(CI)*** | ***M(CI)*** | ***M(CI)*** | ***M(CI)*** | ***λ*** | **F** | ***P*** | **η_p_^2^** |  |  |
| ABAAAQ (Avoid) | 9.51  (7.29-11.74) | 14.8  (13.29-16.31) | 15.97  (13.43-18.51) | 16.29  (14.62-17.95) |  | 8.47 | **<.001** | .10 | b = c = d > a |  |
| ABAAAQ (Approach) | 8.82  (6.69-10.95) | 14.52  (13.08-15.96) | 17.23  (14.81-19.66) | 21.49  (19.90-23.08) |  | 31.81 | **<.001** | .30 | a < b = c < d |  |
| Mean age | 22.32  (21.06-23.57) | 20.95  (20.01-21.90) | 21.73  (19.63-23.83) | 19.94  (18.97-20.92) |  | 2.79 | **.04** | .04 | b = c; d < a |  |
| Age of NSSI onset | 14.67  (13.50-15.83) | 14.81  (14.20-15.42) | 14.17  (13.01-15.32) | 13.16  (12.51-13.80) |  | 4.37 | **.005** | .06 | a = c; a = b > d |  |
| **Mini IPIP** |  |  |  |  | .86 | 2.24 | **.01** | .05 |  |  |
| Extraversion | 12.28  (11.05-13.52) | 10.99  (10.15-11.82) | 10.03  (8.63-11.44) | 9.03  (8.11-9.95) |  | 6.59 | **<.001** | .08 | a = b; a > c > d |  |
| Agreeableness | 16.10  (15.18-17.02) | 16.54  (15.92-17.16) | 16.77  (15.72-17.81) | 15.91  (15.23-16.60) |  | .92 | .43 | .01 | a = b = c = d |  |
| Conscientious | 12.82  (11.76-13.88) | 12.40  (11.68-13.12) | 12.57  (11.36-13.78) | 11.66  (10.87-12.45) |  | 1.25 | .29 | .02 | a = b = c = d |  |
| Neurotic | 13.80  (12.80-14.79) | 14.54  (13.87-15.21) | 15.47  (14.33-16.60) | 15.94  (15.20-16.68) |  | 4.82 | **.003** | .06 | a = b; a > c > d |  |
| Intellect/Imagine | 15.13  (14.01-16.25) | 15.25  (14.49-16.01) | 15.50  (14.22-16.78) | 15.21  (14.38-16.05) |  | .07 | .98 | .001 | a = b = c = d |  |
| **Intrapersonal Functions** |  |  |  |  | .75 | 4.37 | **<.001** | .09 |  |  |
| Affect regulation | 6.82  (6.37-7.27) | 7.64  (7.33-7.94) | 7.73  (7.22-8.25) | 8.31  (7.98-8.65) |  | 9.35 | **<.001** | .11 | a < b = c < d |  |
| Self-punishment | 6.74  (6.15-7.34) | 7.04  (6.63-7.44) | 7.40  (6.72-8.08) | 7.57  (7.13-8.02) |  | 1.99 | .12 | .03 | a = b = c < d |  |
| Anti-dissociation | 5.10  (4.47-5.69) | 5.95  (5.54-6.37) | 6.37  (5.67-7.06) | 6.86  (6.40-7.31) |  | 7.56 | **<.001** | .09 | a < b < c = d |  |
| Anti-suicide | 4.00  (3.36-4.64) | 5.02  (4.59-5.46) | 6.07  (5.34-6.80) | 6.44  (5.97-6.92) |  | 14.40 | **<.001** | .16 | a < b < c < d |  |
| Marking distress | 5.31  (4.68-5.94) | 5.47  (5.04-5.90) | 6.40  (5.68-7.12) | 6.03  (5.56-6.50) |  | 2.71 | **.046** | .04 | a = b; a < c = d |  |
| **Interpersonal Functions** |  |  |  |  | .84 | 1.57 | **.04** | .06 |  |  |
| Interpersonal boundaries | 4.18  (3.66-4.70) | 4.11  (3.75-4.46) | 4.20  (3.61-4.80) | 3.89  (3.50-4.28) |  | .42 | .74 | .01 | a = b = c = d |  |
| Self-care | 4.56  (4.00-5.120 | 4.51  (4.13-4.89) | 5.47  (4.83-6.11) | 5.29  (4.87-5.70) |  | 3.98 | **.01** | .05 | a = b < c = d |  |
| Sensation seeking | 4.00  (3.53-4.47) | 3.96  (3.65-4.28) | 4.23  (3.70-4.77) | 4.30  (3.95-4.65) |  | .79 | .50 | .01 | a = b = c = d |  |
| Peer bonding | 3.31  (3.05-3.57) | 3.41  (3.24-3.59) | 3.10  (2.81-3.40) | 3.17  (2.98-3.36) |  | 1.64 | .18 | .02 | a = b = c = d |  |
| Interpersonal influence | 3.95  (3.50-4.40) | 4.07  (3.77-4.37) | 4.43  (3.92-4.94) | 3.99  (3.65-4.32) |  | .83 | .48 | .01 | a = b = c = d |  |
| Toughness | 4.51  (3.67-5.06) | 4.45  (4.08-4.82) | 4.57  (3.94-5.20) | 4.50  (4.09-4.91) |  | .04 | .99 | .001 | a = b = c = d |  |
| Revenge | 3.33  (3.01-3.65) | 3.56  (3.35-3.78) | 3.60  (3.23-3.97) | 3.31  (3.08-3.55) |  | 1.16 | .33 | .02 | a = b = c = d |  |
| Autonomy | 3.90  (3.42-4.48) | 3.91  (3.58-4.23) | 3.87  (3.32-4.42) | 4.16  (3.80-4.52) |  | .48 | .70 | .01 | a = b = c = d |  |
| **Reasons to Stop** |  |  |  |  | .72 | 2.77 | **<.001** | .11 |  |  |
| Sit & Env Deterrents | 8.54  (7.31-9.77) | 8.49  (7.66-9.33) | 7.70  (6.30-9.10) | 8.34  (7.43-9.26) |  | .35 | .79 | .01 | a = b = c = d |  |
| Neg physical | 10.72  (9.40-12.04) | 9.89  (9.00-10.79) | 10.20  (8.70-11.70) | 10.79  (9.80-11.77) |  | .71 | .55 | .01 | a = b = c = d |  |
| Fear stigma | 12.97  (11.65-14.30) | 12.80  (11.91-13.70) | 12.83  (11.33-14.34) | 13.13  (12.14-14.12) |  | .09 | .97 | .001 | a = b = c = d |  |
| Addiction | 12.13  (10.60-13.66) | 13.54  (12.50-14.58) | 14.43  (12.69-16.18) | 16.02  (14.94-17.23) |  | 6.41 | **<.001** | .08 | b=c; a = b < d |  |
| Others Exp. | 10.33  (9.02-11.64) | 9.91  (9.02-10.79) | 8.97  (7.47-10.46) | 10.56  (9.58-11.54) |  | 1.12 | .34 | .02 | a = b = c = d |  |
| Desire for change/resolve distress | 21.36  (19.45-23.27) | 19.66  (18.37-20.95) | 19.77  (17.59-21.95) | 15.61  (14.19-17.04) |  | 9.59 | **<.001** | .12 | a = b = c < d |  |
| Neg Emotion | 13.05  (11.73-14.37) | 12.78  (11.88-13.67) | 11.87  (10.36-13.37) | 10.90  (9.92-11.88) |  | 3.38 | **.02** | .04 | a = b = c = d |  |
| Neg impact relationships | 17.67  (16.17-19.16) | 16.81  (15.80-17.83) | 16.23  (14.53-17.94) | 15.86  (14.74-16.97) |  | 1.35 | .26 | .02 | a = c; a = b > d |  |
| Body concerns | 13.85  (12.46-15.23) | 12.40  (11.46-13.34) | 12.13  (10.55-13.72) | 10.79  (9.75-11.82) |  | 4.27 | **.01** | .06 | b=c; a = b < d |  |
| **DERS/K10** |  |  |  |  | .66 | 4.29 | **<.001** | .13 |  |  |
| DERS Aware | 6.95  (6.10-7.80) | 7.24  (6.66-7.83) | 7.11  (6.09-8.14) | 9.89  (9.21-10.56) |  | 15.19 | **<.001** | .18 | a = b = c < d |  |
| DERS Clarity | 7.51  (6.47-8.56) | 9.08  (8.37-9.80) | 7.89  (9.74-12.04) | 11.03  (10.20-11.86) |  | 11.17 | **<.001** | .14 | a < b = c < d |  |
| DERS Goals | 11.13  (10.17-12.08) | 11.24  (10.59-11.90) | 10.89  (9.74-12.04) | 12.29  (11.53-13.05) |  | 2.18 | .09 | .03 | a = b = c = d |  |
| DERS Impulse | 6.10  (5.03-7.17) | 7.10  (6.36-7.83) | 6.33  (5.05-7.62) | 8.79  (7.94-9.64) |  | 6.46 | **<.001** | .09 | a = b = c < d |  |
| DERS Non-Acceptance | 8.62  (7.59-9.64) | 9.15  (8.44-9.85) | 8.89  (7.65-10.12) | 11.13  (10.32-11.94) |  | 6.75 | **<.001** | .09 | a = b = c < d |  |
| DERS Strategies | 7.82  (6.94-8.70) | 8.95  (8.35-9.56) | 9.37  (8.31-10.43) | 10.87  (10.17-11.57) |  | 10.56 | **<.001** | .13 | a < b = c < d |  |
| K10 | 27.51  (25.21-29.82) | 31.02  (29.44-32.62) | 30.63  (27.86-33.40) | 35.38  (33.57-37.20) |  | 10.10 | **<.001** | .13 | a < b = c < d |  |
| **NEQ/SERN** |  |  |  |  | 6.23 | 4.56 | **<.001** | .15 |  |  |
| NEQ Affect | 10.74  (9.75-11.74) | 12.47  (11.79-13.15) | 13.60  (12.46-14.74) | 14.71  (13.97-15.46) |  | 14.61 | **<.001** | .17 | a < c = b = d |  |
| NEQ Social | 11.36  (10.13-12.59) | 13.14  (12.31-13.98) | 12.37  (10.96-13.77) | 13.49  (12.57-14.41) |  | 2.83 | **.04** | .04 | a < b = c = d |  |
| NEQ Comm | 8.69  (7.75-9.64) | 8.37  (7.73-9.01) | 8.17  (7.01-9.24) | 7.64  (6.94-8.35) |  | 1.24 | .30 | .02 | a = b = c = d |  |
| NEQ Pain | 15.74  (14.84-16.65) | 14.87  (14.26-15.49) | 15.50  (14.47-16.53) | 14.66  (13.98-15.33) |  | 1.55 | .20 | .02 | a = b = c = d |  |
| NEQ Neg Self | 13.56  (12.51-4.62) | 13.88  (13.17-14.60) | 14.83  (13.63-16.04) | 14.27  (13.49-15.06) |  | 1.00 | .40 | .01 | a = b = c = d |  |
| SERN Risk | 18.56  (16.61-20.52) | 17.44  (16.11-18.76) | 19.93  (17.70-22.17) | 13.47  (12.01-14.93) |  | 10.60 | **<.001** | .13 | a = b = c > d |  |
| SERN Protect | 25.54  (24.79-28.29) | 26.33  (25.14-27.51) | 27.63  (25.64-29.63) | 22.60  (21.29-23.91) |  | 8.92 | **<.001** | .11 | a = b = c > d |  |
| SERN Reminders | 24.54  (22.44-26.64) | 21.81  (20.39-23.24) | 22.60  (20.20-25.00) | 16.19  (14.62-17.76) |  | 16.64 | **<.001** | .19 | a = b = c > d |  |
| *Note:* Significant *p* values are bolded. *M* = Estimated marginal means | | | | | | | | | | |
