# Supplementary material for: Profiling ambivalence in the context of nonsuicidal self‐injury
Source: J Clin Psychol. 2023 Feb 25;79(8):1699–712. doi: 10.1002/jclp.23494 (PMC10952785; doi:10.1002/jclp.23494)
Supplement: Supplementary file 3 — Supporting information. [file JCLP-79-1699-s001.docx]

| *Table S3. NSSI characteristics within profiles* | | | | | | | | |
| --- | --- | --- | --- | --- | --- | --- | --- | --- |
|  | **Avoid**^a^ |  | **Moderate Ambivalence**^b^ |  | **High Ambivalence**^c^ |  | **Approach**^d^ |  |
|  | % Within Profile | SR | % Within Profile | SR | % Within Profile | SR | % Within Profile | SR |
| **Have you wanted to self-injure in your lifetime?** | | | | | | | | |
| Yes | 97.4% | -.1 | 100% | .0 | 100% | .0 | 100% | .0 |
| No | 2.6% | 2.0 | 0% | .0 | 0% | .0 | 0% | .0 |
| **Have you self-injured in the last year?** | | | | | | | | |
| Yes | 56.4% | -1.5 | 79.5% | .2 | 69% | -.5 | 89.7% | 1.2 |
| No | 43.6% | 2.7 | 20.5% | -.4 | 31% | .9 | 10.3% | -2.2 |
| **Have you wanted to self-injure in the last year?** | | | | | | | | |
| **Yes** | 60.5% | -1.5 | 82.4% | .0 | 83.3% | .0 | 94.3% | 1.1 |
| **No** | 39.5% | 3.2 | 17.6% | .0 | 16.7% | -.1 | 10.3% | -2.4 |
| **Have you self-injured in the last month?** | | | | | | | | |
| Yes | 22.2% | -2.1 | 42.0% | -.6 | 37.9% | -.7 | 67.1% | 2.6 |
| No | 77.8% | 2.0 | 58.0% | .5 | 62.1% | .6 | 32.9% | -2.4 |
| **Have you wanted to self-injure in the last month?** | | | | | | | | |
| Yes | 43.5% | -1.7 | 60% | -1.4 | 84% | .6 | 97% | 2.1 |
| No | 56.5% | 2.9 | 40% | 2.4 | 16% | -.9 | 3% | -3.6 |
| **Do you experience pain when you self-injure?** | | | | | | | | |
| Yes | 94.9% | -.2 | 97.6% | .0 | 100% | .1 | 97.1% | .0 |
| No | 5.1% | .9 | 2.4% | -.2 | 0% | -.9 | 2.4% | .1 |
| *Note:* Percentages are within groups; SR = Standardised residual; Standardised residuals lower or greater than 2 indicate significant differences | | | | | | | | |
